# Changthangi Pashmina Goat Genome: Sequencing, Assembly, and Annotation

**DOI:** 10.3389/fgene.2021.695178

**Published:** 2021-07-20

**Authors:** Basharat Bhat, Nazir A. Ganai, Ashutosh Singh, Rakeeb Mir, Syed Mudasir Ahmad, Sajad Majeed Zargar, Firdose Malik

**Affiliations:** ^1^Division of Animal Biotechnology, Sher-e-Kashmir University of Agricultural Sciences and Technology of Kashmir, Srinagar, India; ^2^Department of Life Science, Shiv Nadar University, Greater Noida, India; ^3^Department of Biotechnology, Baba Ghulam Shah Badshah University, Rajouri, India; ^4^Division of Plant Biotechnology, Sher-e-Kashmir University of Agricultural Sciences Technology of Kashmir, Srinagar, India; ^5^Division of Temperate Sericulture, Sher-e-Kashmir University of Agricultural Sciences and Technology of Kashmir, Srinagar, India

**Keywords:** Pashmina goat, whole genome sequence, goat SNP, Pashmina fiber, cold stress

## Abstract

Pashmina goats produce the world's finest and the most costly animal fiber (Pashmina) with an average fineness of 11–13 microns and have more evolved mechanisms than any known goat breed around the globe. Despite the repute of Pashmina goat for producing the finest and most sought-after animal fiber, meager information is available in the public domain about Pashmina genomics and transcriptomics. Here we present a 2.94 GB genome sequence from a male Changthangi white Pashmina goat. We generated 294.8 GB (>100X coverage) of the whole-genome sequence using the Illumina HiSeq 2500 sequencer. All cleaned reads were mapped to the goat reference genome (2,922,813,246 bp) which covers 97.84% of the genome. The Unaligned reads were used for *de novo* assembly resulting in a total of 882 MB non-reference contigs. *De novo* assembly analysis presented in this study provides important insight into the adaptation of Pashmina goats to cold stress and helps enhance our understanding of this complex phenomenon. A comparison of the Pashmina goat genome with a wild goat genome revealed a total of 2,823 high impact single nucleotide variations and small insertions and deletions, which may be associated with the evolution of Pashmina goats. The Pashmina goat genome sequence provided in this study may improve our understanding of complex traits found in Pashmina goats, such as annual fiber cycling, defense mechanism against hypoxic, survival secret in extremely cold conditions, and adaptation to a sparse diet. In addition, the genes identified from *de novo* assembly could be utilized in differentiating Pashmina fiber from other fibers to avoid falsification at marketing practices.

## Background

The domestic goat (*Capra hircus*) is an Asian animal distributed across all ecologies ranging from cold arid to hot humid. It serves as an important source of meat, milk, skin, fiber, and manure. Goats exhibit several traits and diseases that are similar to those of humans, which is the reason why goats are being extensively used as an animal model for biomedical research (Fulton et al., 1994). Modern domestic goats have been domesticated from *Capra aegagrus* (Dong et al., 2015; Sheikh et al., 2016). Over centuries, different goat breeds have been established through evolution and genetic selection, exhibiting traits such as (1) coat color variations, (2) adaptability to different climatic conditions, (3) change in size, (4) development of fine fiber (Dong et al., 2015).

An important breed of extremely cold Temperate Himalayan region of India is Changthangi Pashmina goat which produces world's most sought-after natural animal fiber, **Pashmina** (Bhat B. et al., 2019). These goats are also referred as Pashmina goats or Cashmere goats. Pashmina goats are double hair coated with an outer coat of long coarse guard hair and an inner coat of shorter fine Pashmina fiber. The guard hair develops from primary hair follicles (PHFs) and Pashmina from secondary hair follicles (SHF) (Ansari-Renani et al., 2011). These goats survive in extreme climatic conditions [+35^*o*^C (short summer) and −40^*o*^C (long winter)] at an altitude of 4,000–5,500 m above mean sea level (AMSL) under cold and hypoxic conditions. To adapt to the harsh climatic conditions like cold, hot, arid, dry, and poor grazing conditions, these goats have acquired many special abilities and attributes. The growth of a double hair coat in Pashmina goats may be one of the mechanisms of protection from cold, and the result of the response triggered by the thermoregulatory center of the brain.

It is believed that the Pashmina goats were originated in the Himalayan ranges and migrated to different regions of central Asia ranging from China, Mongolia, Iran, Russia, Afghanistan, and India (Ryder, 1966). China is the leading Pashmina producers followed by Mongolia, Iran, New Zealand, and Britain. Even though India—Kashmir contributes <1% of the world's Pashmina production, because of its fineness and quality it holds a unique position in the world's Pashmina trade (Shakyawar et al., 2013). Globally Pashmina is sold under the geographical indication (GI) tract of *Cashmere*, which belongs to India—**Kashmir**. Changthangi Pashmina goats produce the world's finest and the most costly Pashmina fiber with an average fineness of 11–13 microns (Bumla et al., 2012).

Pashmina fiber is the primary source of income for the nomadic population of Ladakh (Sheikh et al., 2016). Apart from the economic value of the Pashmina goat, this goat serves as an important source of food (milk and meat), skin, and manure in the region. This goat is a safe and secure form of investment and stable means of income in the cold and arid deserts of Ladakh. These animals often provide the only practical means of utilizing vast areas of natural grasslands in the areas where crop production is uneconomical. Adapted to the harsh environmental conditions, the Changthangi goat is a unique genetic resource of the country. However, due to lack of breeding policy and population structure over a long period of time, it is suspected that inbreeding in these indigenous breeds may pose serious threat (Ganai et al., 2011).

In this study, we report the first Pashmina goat genome sequence. We generated 294.8 GB (>100X coverage) raw reads from a Changthangi Pashmina goat using Illumina HiSeq 2500 sequencer. We deciphered 2.94 GB of the Pashmina goat genome, revealing 26,687 protein-coding genes, 842 miRNAs, 188 lncRNAs, and 1879 snRNAs. We compared the Pashmina goat genome with the wild goat genome revealed important genetic variants related to the evolution of Pashmina goats.

## Methods

### Ethics Statement

This study was approved by the Institutional Animal Welfare and Ethics Committee of the Sher-e-Kashmir University of Agricultural Science and Technology of Kashmir (SKUAST-K). All experiments and methods were performed in accordance with relevant guidelines and regulations. Experimental goats were housed in SKUAST-Kashmir goat farm located in the northern Himalayas (Satakna, Ladakh) at an altitude of 5,000 m AMSL.

### Sampling, Genome Sequencing, and Assembly

Genomic DNA was extracted from the blood of a 26 months old male Changthangi Pashmina goat. A whole-genome sequencing (WGS) library was prepared with the Illumina-compatible NEXTflex Rapid DNA sequencing kit (BIOO Scientific, Austin, Texas, U.S.A.) as per the manufacturer's guidelines. Genomic DNA was sheared using Covaris S2 sonicator (Covaris, Woburn, Massachusetts, USA) to generate approximate fragment size distribution from 200 to 400 bp. Here, the fragment size distribution was checked on Agilent Bioanalyzer and subsequently purified using Hiprep magnetic beads (Magbio). Purified fragments were end-repaired, adenylated, and ligated to Illumina multiplex barcode adaptors as per NEXTFlex Rapid DNA sequencing kit protocol. Adapter-ligated DNA was purified and size selected using Hiprep beads. Resultant fragments were amplified for four cycles of PCR using Illumina-compatible primers provided in the NEXTFlex Rapid DNA sequencing kit. The final PCR product (i.e., sequencing library) was purified with Hiprep beads, followed by a library-quality control check. Illumina compatible sequencing library was initially quantified by Qubit fluorometer (Thermo Fisher Scientific, MA, USA) and its fragment size distribution was analyzed on Agilent TapeStation. Sequencing and base calling were performed according to the Illumina recommendations.

Using the Illumina HiSeq 2500 platform, a total of 294.8 GB (150 bp reads) high-quality data (1~00X coverage of the estimated genome size) were generated. The raw reads were pre-processed to remove the adapter sequences, low-quality reads, and low-quality bases filtration toward 3′- end using cutadapt program v3.1 (Martin, 2011). Filtered reads were mapped to *C. hircus* reference genome assembly ARS1 downloaded from National Center for Biotechnology Information (NCBI) using bowtie2 v2.4.2 (Langmead and Salzberg, 2012). The Unmapped paired-end reads to reference were used for contig assembly using Abyss *de novo* assembler v2.0 (Jackman et al., 2017). The complete assembly was obtained by merging the reference-assisted and *de novo* assembled consensus sequences. The completeness and correctness of genome-assembly were evaluated with the Benchmarking Universal Single-Copy Orthologs (BUSCO) program v5. 1.2 (Simão et al., 2015).

### Genome Annotation

The repetitive elements were identified in the final assembled draft genome using RepeatMasker v4.0.9 (Tarailo-Graovac and Chen, 2009). The transfer RNAs (tRNAs) were predicted using the tRNAScan-SE program v2 (Lowe and Chan, 2016), with default parameters for eukaryotic genomes. Ribosomal RNAs (rRNAs) were collected based on homology information from *Homo sapiens, Bos taurus, Bos mutus* and *Ovis aris* rRNAs using the BlastN program v2.11.0+ (Ye et al., 2006). Other non-coding RNA were predicted from the assembled genome with Infernal v1.1.2 (Nawrocki and Eddy, 2013) using the Rfam database v13 (Griffiths-Jones et al., 2003), with an *E*-value cutoff of 0.001. Only non-truncated CM hits detected in the first pass of the pipeline with an inclusion threshold of ≤ 0.001 and score ≥45 were reported. Overlapping hits with lower score hits were filtered out.

Protein-coding gene identification was performed using Augustus (Stanke and Waack, 2003) and Maker v2.31.10 (Cantarel et al., 2008) programs. A total of 26,687, protein-coding genes were identified using a 2-pass schema. The first round of gene prediction was carried out using AUGUSTUS with *H. sapiens, B. taurus, B. mutus*, and *O. aris* as reference models for the hard-masked assembled genome. The predicted gene model from AUGUSTUS was taken as input along with transcripts and the available gene model of *C. hircus* for the second round of gene prediction in the MAKER tool. The MAKER tool provides evidence-based gene modeling. After performing the gene prediction through MAKER, we selected the following filtering criteria for the selection of the final gene model.

- Predicted gene should have a start and stop codon.
- Length of the gene should be >300 bps.
- Gene does not contain more than 1% of N's.

Predicted proteins were annotated using homology-based prediction by searching against mammalian gene sequences utilizing the BLAST program.

- At first the protein sequences were similarity searched against the UniProt Bovidae (91,402) and Caprine family (91,402) protein database using BLASTP program with an *e*-value of 0.00001 for gene ontology (GO) and annotation (Consortium, 2014; Shaik et al., 2019).
- The unmapped genes were homology searched against NCBI *C. hircus* (GCF-001704415.1) proteins (42,687) using the BLASTP program with an *E*-value cut-off of 0.00001.
- The unannotated sequences were further annotated against NCBI non-redundant (NR) database and Pfam database with default parameters. A total of 90% of predicted genes were annotated.

The predicted proteins were uploaded to the KEGG (Kyoto Encyclopedia of Genes and Genomes)—KAAS (KEGG Automatic Annotation Server) (Moriya et al., 2007) server for pathway identification using *B. taurus, B. mutus, C. hircus, Bos indicus* and *Ovis aries* as reference organisms.

### Variant Detection and Annotation

Variant identification was done using the Genome Analysis Toolkit (GATK) v4.0.7.0 (McKenna et al., 2010) applied on bowtie output. As recommended by the GATK practice, Picard tools v2.25.1 (Pic, 2019) were used to add read group information, mark duplicates, and index a sorted BAM file. As per the GATK pipeline following steps were performed for variant calling from genomic data; split and trim to reassign mapping quality, local realignment, InDel realignment, and BaseQualityScore recalibration. For variant discovery, HaplotypeCaller and GenotypeGVCFs were used followed by filtering variants. Identified variants were divided into different functional classes based on their genomic distribution. *C. hircus* gene annotation file was used to determine if a SNP is located within mRNA start and end positions (genic), CDS, 5′UTR, or 3′UTR. The variants identified were filtered for the coverage of more than 20 (more than 20 reads covering the position) and quality of 30 (Phred scaled quality of more than 30) to include high confidence variants. Variants were annotated using SnpEff program v4.3T (Cingolani et al., 2012). To further evaluate the biological significance of the genes with high impact variations, the pathway analysis were performed using KEGG-KAAS server (Moriya et al., 2007; Shaik et al., 2019).

## Results and Discussion

### Genome Sequence and Annotation

We sequenced genome DNA from a 26-month-old male Changthangi Pashmina goat. High-quality DNA extracted from blood was used to construct paired-end sequencing libraries. Using the Illumina HiSeq 2500 platform, a total of 294.8 GB raw data were generated. Out of the total cleaned reads (965,981,627 reads), 80% reads were mapped to the reference genome, covering 97.84% genome. To identify genes specific to Pashmina goats, *de novo* assembly of the unaligned reads were performed. The final genome assembly was obtained by merging the reference-assisted and *de novo* assembled consensus sequences. The final gap closer was executed using GapCloser program (Simpson and Durbin, 2012) with PE-LI libraries which generated a final draft genome of 2.94 GB with an N50 value of 102582650 (Supplementary Table 1). Calculation of the completeness of genome assembly using BUSCO program suggested 99.3% of the assembled genome was complete (85.6% complete and single-copy, 13.7% complete and duplicated) remaining 0.7% of genome were fragmented.

A total of 18,656 contigs (Supplementary Table 1) were assembled from *de novo* assembly, which may be specific to the Pashmina goat breed. To interpret the biological implication of sequences extracted from *de novo* assembly, all sequences were mapped to the KEGG database using KASS servers (using cut-off FDR corrected *q*-value < 0.01). KEGG pathways analysis deduced that the sequences are predicted to be involved in metabolism pathways like amino-acids (cysteine, methionine, threonine, serine, and glycine), nucleotides metabolism, fatty acid metabolism; and signaling pathways like Calcium signaling, Notch signaling, cAMP signaling, Rap1, and PI3K-Akt signaling. Amino-acids and energy play a vital role in continuous Pashmina fiber growth and follicle initiation. Enrichment analysis suggests the potential role of cAMP, PI3K-Akt, and calcium signaling in regulating cold stress responses in Pashmina goats. Further transcriptomic or proteomic studies are required to identify specific genes and pathways mediating cold stress response in Pashmina goats.

A total of 1,450,391,539 bp (49.30% of the genome) of the Pashmina goat genome contain repetitive elements (Table 1) which is in accordance with the earlier studies of cattle (*B. taurus*) genome (50%) (Sequencing et al., 2012). In our study, we identified that long interspersed elements cover 25.37% of the whole genome, which is equitable to that of cattle (23%), human (21%), and horse (20%) (Sequencing et al., 2012). The percentage of short interspersed elements is 10.07% which is lower than that of humans (13%) and cattle's (18%) and greater than that of mice (8%) and horses (7%). 40.83% of the total Pashmina goat genome contains different types of interspersed repeats. Also, 8.44% of the total Pashmina genome contains small RNAs, microsatellites, and simple repeats; which should be useful in quantitative trait locus mapping or marker-assisted breeding in modulating economically important traits in specialty animal fiber like Pashmina.

**Table 1:** Repeat element statistics from Pashmina goat genome assembly.

	**No. of elements**	**Length occupied (bp)**	**%age in genome**
SINEs	2,045,717	296,111,061	10.07
MIRs	391,052	55,830,025	1.90
LINEs	1,316,415	746,218,268	25.37
LINE1	567,547	331,707,256	11.28
LINE2	246,431	61,914,088	2.10
L3/CR1	33,442	6,797,367	0.23
RTE	467,971	345,644,042	11.75
LTR elements	349,139	102,247,773	3.48
ERVL	72,022	28,212,673	0.96
ERVL-MaLRs	120,773	39,051,305	1.33
ERV_classI	39,090	13,144,887	0.45
ERV_classII	101,109	18,070,827	0.61
DNA elements:	278,549	55,919,821	1.90
hAT-Charlie	160,945	29,848,276	1.01
TcMar-Tigger	43,361	11,562,004	0.39
Unclassified	4,534	803,290	0.03
Small RNA	250,244	39,202,683	1.33
Satellites	137,840	149,989,331	5.10
Simple repeats	823,810	89,185,768	3.03
Low complexity:	105,341	8,887,201	0.30
Total repeats:		1,450,391,539 bp	49.30

A multi-way approach was carried out for protein-coding gene prediction from the final assembled genome. We have used AUGUSTUS and MAKER tools for gene prediction. The first round of gene prediction was carried out using AUGUSTUS with humans as a reference model for the hard-masked assembled genome. The predicted gene model from AUGUSTUS was taken as input along with transcripts and the available gene model of *C. hircus* for the second round of gene prediction in the MAKER tool. A total of 26,687 protein-coding genes were identified by combining reference and *de novo* assembled genome (Supplementary Table 2).

### Variant Detection

GATK workflow identified 14,270,872 SNVs. The transitions to transversions (Ts/Tv) ratio was 2.35 and homozygosity (4,965,247) and heterozygosity (9,306,235) percentage were 34.79 and 65.21%, respectively, both in accordance with the earlier studies of mammalian genome analysis. Of the SNPs, 35% are intronic, 63% intergenic, 0.6% were in 3′ and 5′ UTR regions. 206,385 SNVs were identified in coding regions with 96,092 as synonymous and 50,355 as non-synonymous variants. Distributions of SNVs and InDels in different chromosomes of the Pashmina goat genome are presented in Supplementary Figure 1. A total of 1,423 high-impact SNVs (start lost-103, stop gained-790, and stop lost-530) were also identified which may be associated with the evolution of the Pashmina goat (Supplementary Table 3). We identified 1,126,239 InDels, which consisted of 484,468 insertions and 641,871 deletions. The length distribution of identified InDels ranged from −28 to +28 bp and homozygosity (621,032) and heterozygosity (505,207) percentages were 55 and 45%, respectively. Of all InDels, 61.7 and 37% were in intergenic and intronic regions, respectively. 0.01% InDels were divided between UTR regions of genes. A total of 1,400 high-impact InDels were identified which contained 1,176 frameshift, 20 and 5 stop lost, and stop gained InDels, respectively (Supplementary Table 4).

Functional analysis suggest genes with high impact SNPs were involved mainly in signaling pathway like Notch, MAPK, AMPK, Calcium, PPAR, mTOR, TNF, Neurotrophin, Jak-STAT, VEGF, and Wnt (Table 2). These signaling pathways are involved in diverse biological processes and their specific role in Pashmina goats needs to be further elucidated.

**Table 2:** Pathways affected by high impact SNPs and InDELs in Pashmina goat genome.

**Pathways**	**Genes**
Cytokine-cytokine receptor interaction	BMP3, FAS, LEPR, CCR3, IL3RA, TNFRSF11A, IFNGR2, IL1A, CXCL17
ECM-receptor interaction	COL6A5, TNN, ITGB5, COL4A1
Notch signaling pathway	PTCRA, DLL3
MAPK signaling pathway	RASGRP2, CACNA1I, FAS, MAPK11, IL1A, CACNA1S
AMPK signaling pathway	ACACB, CPT1B, LEPR
Calcium signaling pathway	CACNA1I, ATP2B3, CACNA1S, MYLK
PPAR signaling pathway	CPT1B, PLIN2
mTOR signaling pathway	GRB10, ATP6V1G1, WDR59
Neuroactive ligand-receptor interaction	GRID1, CHRNB3, TAAR8, LEPR, RLN3, C3
Circadian entrainment	PER3, CACNA1I
TNF signaling pathway	FAS, MAPK11
Neurotrophin signaling pathway	IRAK2, MAPK11
Jak-STAT signaling pathway	IL3RA, IFNGR2, LEPR
VEGF signaling pathway	MAPK11
Wnt signaling pathway	APC2, ROR2

### Genome-Wide Identification of lncRNAs

Long non-coding RNAs (lncRNAs) are emerging as key regulators for a myriad of biological processes. In the current lncRNA databases, most of the identified lncRNAs are derived from mice and humans (Volders et al., 2014). Several recent studies on *B. taurus* (Huang et al., 2012), *Gallus gallus* (Li et al., 2012), and *Sus scrofa* (Tang et al., 2017) have increased the information pool of lncRNAs. However, meager information is available on lncRNAs for *Caprines* species. To our knowledge, this is the first study to report genome-wide identification of lncRNAs from any goat species. In this study, we systematically identified a comprehensive list of lncRNA (Supplementary Table 5) identified from the Pashmina goat genome and their role in regulating different biological processes.

Recent studies suggest the role of lncRNAs in modulating immunity; in our study, we identified three major classes of immune regulatory lncRNAs. (1) HOX antisense intergenic RNA myeloid 1 (*HOTAIRM1*) is known to be associated with granulocytes, and is a key regulator in myeloid transcriptional regulation, by modulating *HOXA* expression in cis-configuration (Mumtaz et al., 2017). (2) *HOX* transcript antisense RNA (*HOTAIR*) is an oncogenic long non-coding RNA overexpressed in various carcinomas. It recruits various chromatin-modifying enzymes and regulates gene silencing (Bhan and Mandal, 2015). (3) Nuclear Enriched Abundant Transcript 1 (NEAT1) known to the innate immune response to viral infection (Mumtaz et al., 2017).

LncRNA also plays a critical role in mediating gene expression during different developmental and differentiation processes. We identified six classes of lncRNA, which are known for mediating developmental traits in different animal models. (1) *KCNQ1* overlapping transcript 1 (*KCNQ1OT1*) plays crucial role in the transcriptional silencing of the *KCNQ1* locus by regulating histone methylation. *KCNQ1OT1* gene inactivation results multiple growth defects in mice (Fatica and Bozzoni, 2014). (2) *HOXA* transcript at the distal tip (*HOTTIP*) knockdown of these genes results in retinal cell development in mice and altered limb morphology in chickens (Fatica and Bozzoni, 2014). (3) *H19* has a role in cell proliferation; *H19* limits body growth by regulating *IGF2* expression. Mice with the loss of H19 function show an overgrowth phenotype. (4) Metastasis associated lung adenocarcinoma transcript 1 (*MALAT1*) is majorally involved in neural development, in cultured mice hippocampal neurons *MALAT1* knockdown shows decreased dendritic growth and decreased synaptic density. (5) X-inactive specific transcript (*XIST*) acts as a regulator of X-chromosome inactivating in mammals. *XIST* deletion in mice causes a loss of X-chromosome inactivation and female-specific lethality. (6) Myocardial infarction-associated transcript (*MIAT*) is associated with retinal cell fate and myocardial infarction in humans. Other identified ncRNAs (rRNA, snRNA, tRNA, lncRNAs, and miRNAs) including FASTA sequences are listed in Supplementary Table 5.

### Genome-Wide Identification of miRNAs

MicroRNAs (miRNAs) are small (22 nt long), non-coding regulatory RNAs, which can evoke post-translational repression of mRNA levels of target genes. miRNAs are not only involved in transcriptional/post-transcriptional regulation but also regulate response to environmental stresses. In this study, using high-throughput genome sequencing we identified a total of 338 mature miRNAs in the Changthangi Pashmina goat genome. The length of mature miRNAs varies from 17 to 25 nucleotides with an average of 21 nucleotides. The major class of miRNAs 91% falls within the range of 20–23 nucleotides (Supplementary Figure 2). The highest number of miRNAs are observed in the mir-1255 family followed by mir-1302, mir-544, mir-692, mir-154, mir-562, mir-663, mir-684, mir-650, and let-7.

The miR-1255 commonly express in exosome and regulates TGF-β signaling pathway by interacting with SMAD4 gene (Xin et al., 2020). MicroRNA-214, miR-31 and miR-218 controls skin and hair follicle development by modulating Wnt signaling and β-catenin signaling (Mardaryev et al., 2010; Ahmed et al., 2014; Hu et al., 2020; Bhat et al., 2021). miR-128, miR-148, and miR-301 regulate key genes involved in cholesterol-lipoprotein trafficking (Wagschal et al., 2015). miR-187 family regulates key genes (*TGFB1, THBS1, ACVR18* and *BMP88*) in TGF-beta signaling pathway (Miao et al., 2016; Bhat S. A. et al., 2019). Complete annotation of miRNAs from Pashmina goat genome were listed in Supplementary Table 5.

### Cold Stress Response in Pashmina Goat: A Possible Regime

Cold tolerance in Pashmina goats is an extremely complex phenomenon that is influenced by a large number of physiological, biochemical, and endocrine factors. However, the molecular mechanisms underlying the adaptation to cold stress remain largely unknown. The changes that produce a cold-hardy phenotype under cold tolerance situation would involve acclimation and acclimatization in response to low temperatures, rapid cold-hardening, and cold-induced gene expression (Hansen, 2004).

**Figure 1:**
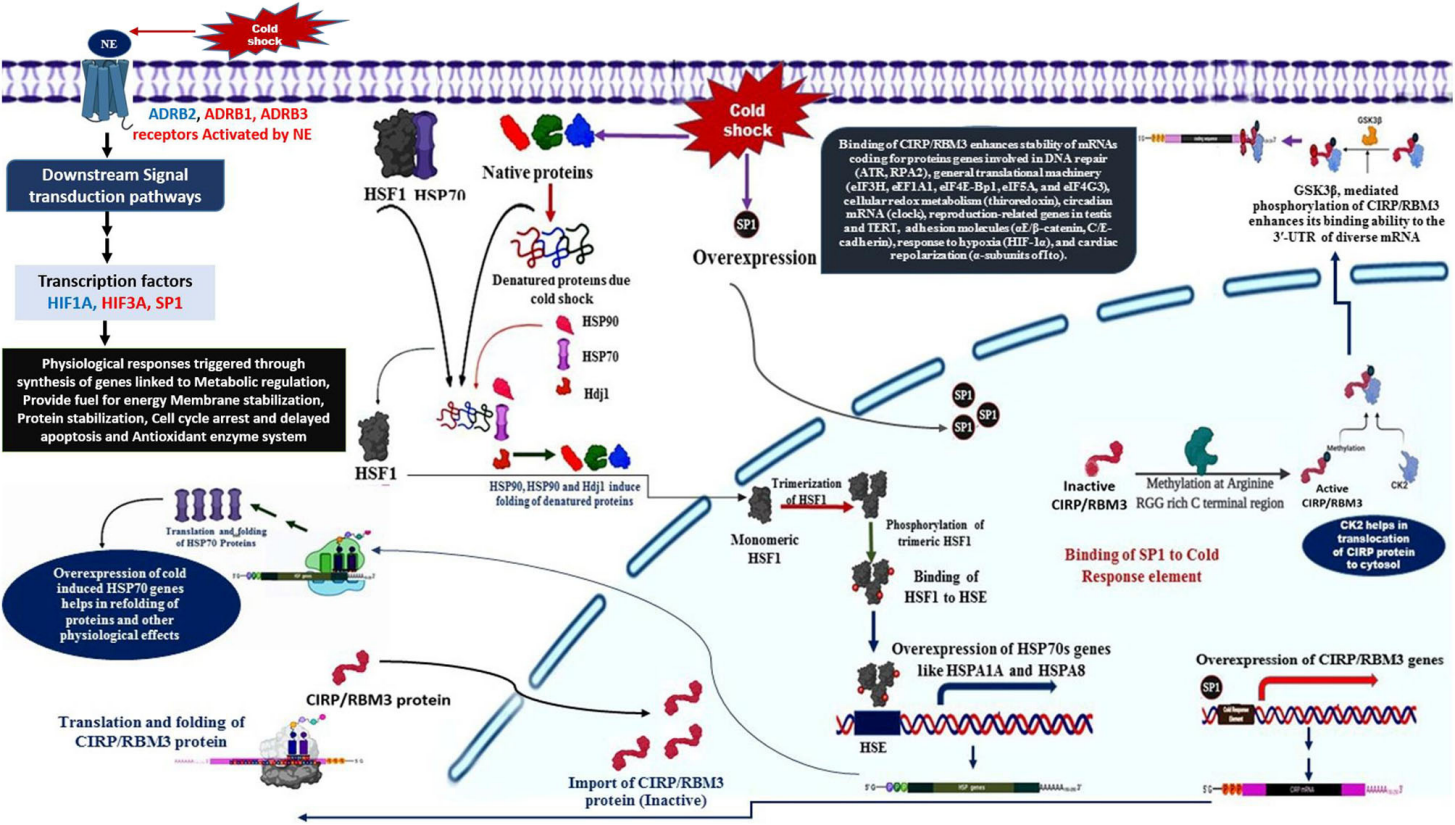
Biological model with assigned role of various cold response genes.

The primary response to cold stress in animals is suggested to be a neuroendocrine response, which triggers the release of catecholamine hormone, usually nor-epinephrine (NE). The cold signal in the form of NE is perceived by beta-2 Adrenergic receptor, a GPCR (Bhat et al., 2017), predominantly localized to the vascular system in comparison to β1 and β3. This signal perception by β*2AR* is known to activate multiple intracellular signal transduction pathways that influence various molecular, biochemical, and physiological processes (Figure 1). NE signaling through β*2AR* regulates blood pressure, heart, and respiratory rate, and body temperature (Chruscinski et al., 2001).

- The cold signal in the form of NE is perceived by β-adrenergic receptors, such as *ADRB1, ADRB2*, and *ADRB3*.
- Activation of β*2AR* triggers several downstream signaling cascades (DSC).
- The DSC lead to the down-regulation of *HIF3A* and also activation of *SP1* and *HIF1A*.
- The activation of SP1 leads to the over-expression of *CIRP* and *RBM3* genes with the help of *CK2* and *GSK3*β (Aoki et al., 2002; Yang et al., 2006; De Leeuw et al., 2007; Sumitomo et al., 2012).
- *CIRP* and *RBM3* proteins are predominantly localized in the nucleus, but can migrate to cytoplasm upon stress condition, and acts as an RNA chaperone regulating mRNA stability through its binding signature site in the 3′-UTR of its targets, which includes genes involved in DNA repair (*ATR, RPA2*), cellular redox metabolism (thiroredoxin), adhesion molecules (αE/β-catenin, C/E-cadherin), circadian mRNA (clock), reproduction-related genes in testis and *TERT*, response to hypoxia (*HIF-1*α), general translational machinery (*eIF3H, eEF1A1, eIF4E-Bp1, eIF5A*, and *eIF4G3*), and cardiac repolarization (α-subunits of Ito). In addition, *CIRP* can also be secreted into extracellular space through lysosome pathway upon stimulation by *LPS* or hypoxia/reoxygenation (Xia et al., 2012).

Another class of protective agents, the heat shock proteins (HSPs), also contribute significantly to the overwintering cold tolerance (Rinehart et al., 2007). Stress factors, like cold, induces over-expression of heat shock genes responsible for the synthesis of molecular chaperones to refold the misfolded of cold-induced proteins. Later mechanism is triggered by the stress-induced synthesis of HSFs, which bind to heat shock elements (HSE) consisting of the pentanucleotide motif *5*′*-nGAAn-3*′ (Lis and Wu, 1993; Tissieres and Georgopoulos, 1994; Voellmy, 1994).

*HSFI and HSP70 interaction in cold-stress response*

- *HSF1* is activated by unfolded proteins resulted due to cold shock, in its inactivated monomeric state *HSF1* is bound to *HSP70* (Santoro, 2000).
- The cold induced unfolded proteins are bound by molecular chaperones, such as *HSP90, HSP70*, and *HDJ1* (Santoro, 2000).
- The released *HSF1* is translocated to nucleus, where they undergo trimerization and phosphorylated (Santoro, 2000).
- The phosphorylated trimeric *HSF1* binds to HSE located upstream of HSP genes, resulting in transcriptional activation and synthesis of HSPs, such as *HSP70, HSPA1A*, and *HSPA8* genes (Banerjee et al., 2014).
- The transcripts of HSPs are shuttled to the cytosol for translation, the higher expression of HSP, in turn, regulate folding and Activation of a specific class of transcription factors called as heat shock factors (HSFs) (Åkerfelt et al., 2010).

Further, in context to the status of the translation process during cold stress condition, it is suggested that the cAMP/PKA pathway is involved in the dysregulation of translation factors like *EIF6, TUFM, EIF1AD, EIF2B2, EIF2B3, RPL7, EEF2, EIF3H*, and *GARS* to modulate the cold stress-responsive protein synthesis. Generally, cold shock results in protein degradation, but an adaptive response is generated to maintain integrity as well as stability of proteins by selective expression of certain heat shock proteins (e.g., *DNAJA4, DNAJB12, DNAJC7, DNAJC11, HSPA8*, and *HSP90B1*).

Cold shock results in changes to the lipid bilayer and composition of the membrane in mammalian cells. The fatty acid and lipid metabolism especially beta-oxidation enzymes (*ACSL1, SLC27A1, ACADVL, HADHA*) are increased to provide fuel in the form *acetyl CoA* for energy generation as well as thermogenesis. Furthermore, HIF1A induced glycolytic enzymes like *G6PD, PFKFB3* are up-regulated, resulting in an increase in energy generation during the hypoxic condition. Thus, glucose metabolism and lipid metabolism are regulated in such a way to meet the requirement of energy while maintaining the stability of the lipid membrane. Cold exposure leads to increased production of reactive oxygen species (ROS) which influence HIFs activity and involves in lipid peroxidation (Quirós et al., 2016). Glutathione peroxidase (GPX), catalase (CAT), and peroxiredoxin (PRDX) are activated to neutralize the ROS effect.

Hence, the cross-talk among various signaling pathways mainly hormonal signaling (NE signaling), cAMP/PKA signaling, Src kinase–PI3K/Akt-dependent pathway, Ca2+ signaling, ERK1/2 signaling, p38 signaling, and ROS signaling could be responsible for the generation of a stress tolerance response in Pashmina goats by modulating the expression of specific cold stress-responsive genes. Figure 1 illustrates the role of various cold-responsive genes.

## Conclusions

Here, we report the characterization of the high altitude Pashmina goat genome for the first time. The present study on the genome and annotations may provide Pashmina breeders and other researchers with useful information regarding trait biology and their subsequent improvement. In particular, we highlight pathways that could be involved in cold-stress response and fiber cycling in Pashmina goats. The annotation of coding and non-coding genes provides, for the first time, an understanding of the gene content in Pashmina goat, which is valuable for future studies on genes, gene structure, and functional genomics.

## Data Availability Statement

The datasets presented in this study can be found in online repositories. The names of the repository/repositories and accession number(s) can be found at: https://www.ncbi.nlm.nih.gov/, SRR7739516.

## Ethics Statement

The animal study was reviewed and approved by Sher-e-Kashmir University of Agricultural Sciences and Technology-Kashmir (Certificate Number: AU/FVSc/PS-57/2058).

## Author Contributions

BB and NG designed the study. AS, BB, SA, and NG planed the work-flow. BB performed data analysis. BB, RM, and SM wrote the manuscript. All authors reviewed the manuscript.

## Conflict of Interest

The authors declare that the research was conducted in the absence of any commercial or financial relationships that could be construed as a potential conflict of interest.
