# Supplementary material for: Changthangi Pashmina Goat Genome: Sequencing, Assembly, and Annotation
Source: Front Genet. 2021 Jul 20;12:695178. doi: 10.3389/fgene.2021.695178 (PMC8329486; doi:10.3389/fgene.2021.695178)
Supplement: Supplementary file 1 [file Image_1.pdf]

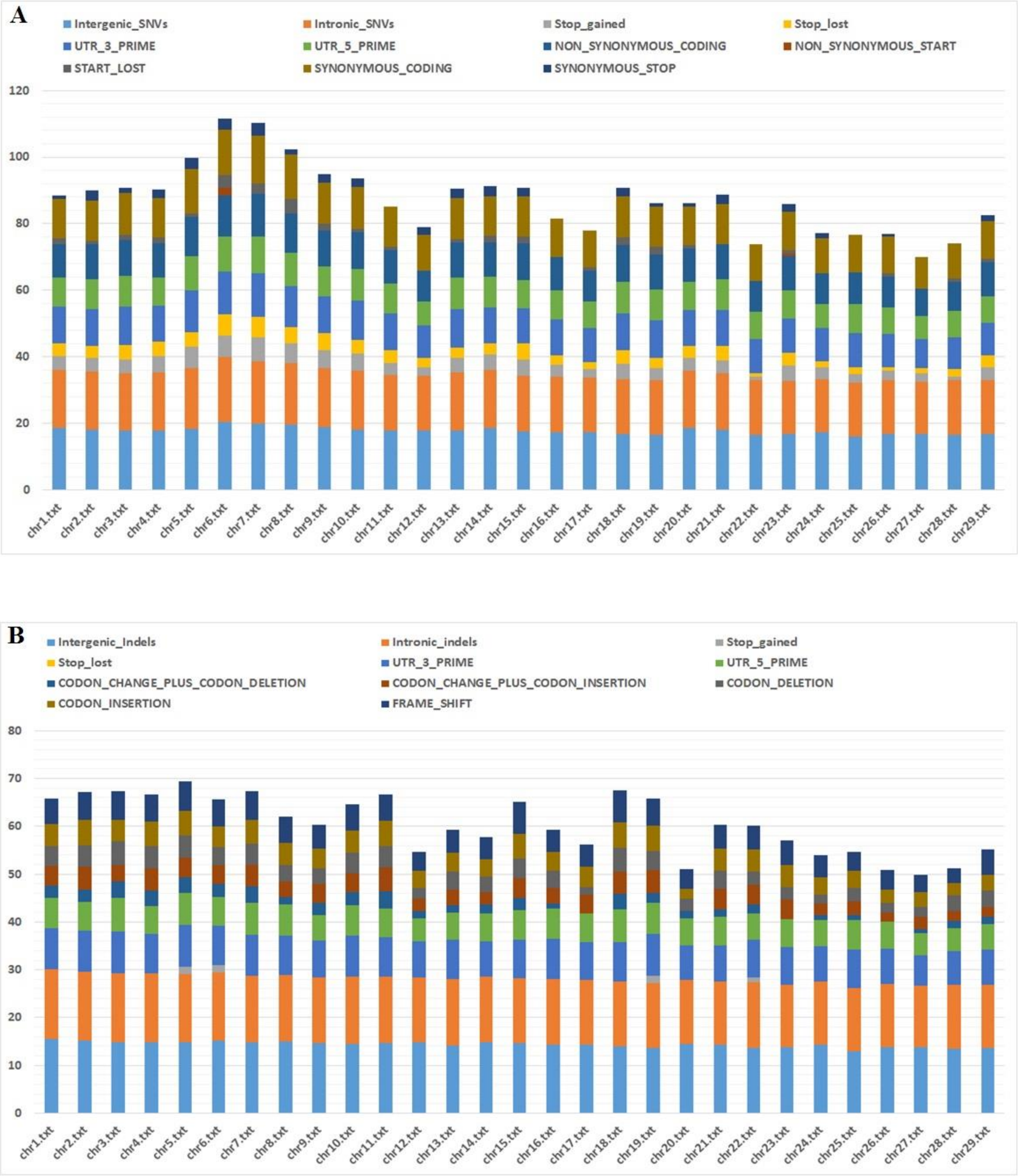

Figure 1. Chromosomal distribution of SNVs (A) and Indels (B) in Changthangi Pashmina goat genome. X-axis represent number of variants (transformed to Log10) and Y-axis represent distributions in different chromosomes
