# Supplementary figures and images for: Changthangi Pashmina Goat Genome: Sequencing, Assembly, and Annotation
Source: Front Genet. 2021 Jul 20;12:695178. doi: 10.3389/fgene.2021.695178 (PMC8329486; doi:10.3389/fgene.2021.695178)

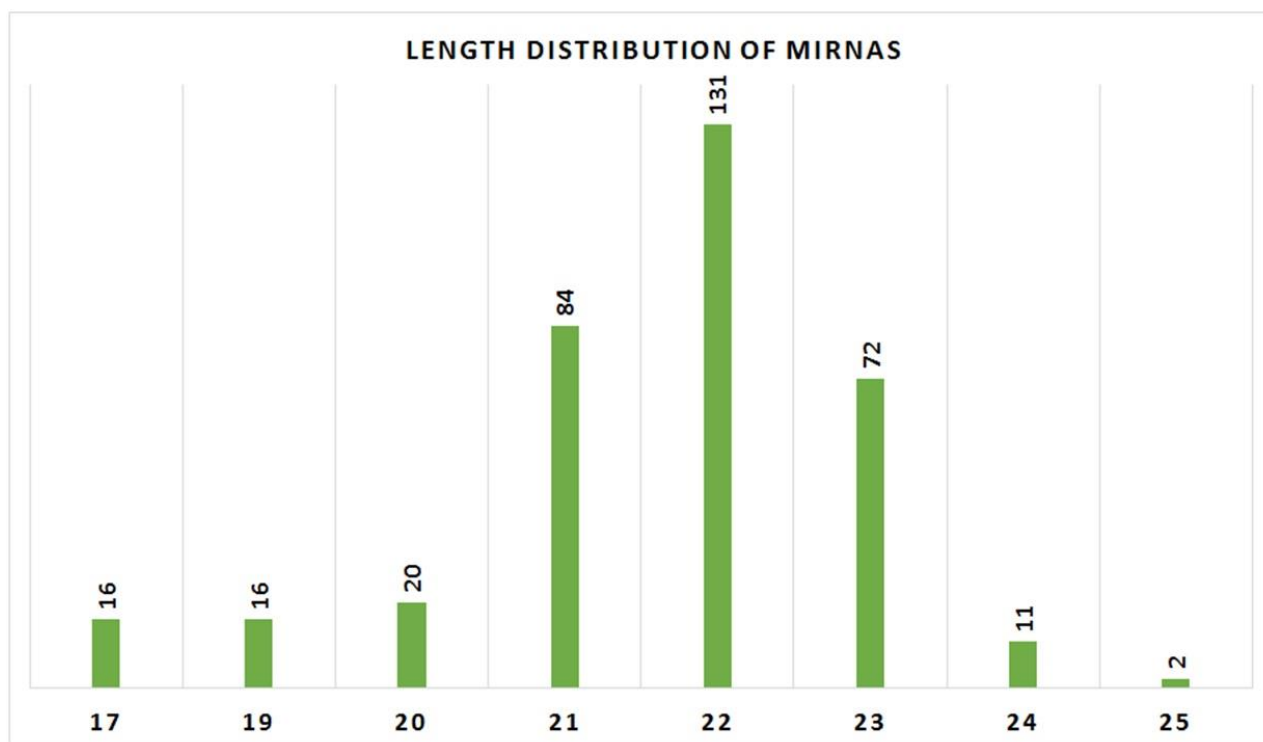

**Figure 2.** Length distribution of mature miRNAs in Pashmina goat genome.

Supplement: Supplementary file 2 [file Image_2.pdf]
