# Supplementary material for: Changthangi Pashmina Goat Genome: Sequencing, Assembly, and Annotation
Source: Front Genet. 2021 Jul 20;12:695178. doi: 10.3389/fgene.2021.695178 (PMC8329486; doi:10.3389/fgene.2021.695178)
Supplement: Supplementary file 3 [file Table_1.pdf]

Table 1. *De novo* and reference assisted genome assembly statistics

	**Reference assisted Scaffolds**	***De novo* Assembly**	**Final Draft Assembly (Gapcloser)**
Contigs Generated	29780	18656	48436
Total Contigs Length (bp)	2.91 GB	882 MB	2.94 GB
Average Contig Length (bp)	247403	5869	198404
Contigs >= 1 Mbp	32	1	33
Total Number of Non-ATGC (bp)	7849819 bp	1284 bp	3275 bp
N50 value	101277228	330986	102582650
